# Effect of Vandetanib Treatment on Cystic Changes in the Liver following Metastasis from Medullary Thyroid Carcinoma

**DOI:** 10.1155/2022/9855403

**Published:** 2022-04-06

**Authors:** Ai Matsui, Soji Toda, Daisuke Murayama, Hiroyuki Iwasaki

**Affiliations:** Department of Breast and Endocrine Surgery, Kanagawa Cancer Center, Yokohama, Japan

## Abstract

A number of causes are responsible for the development of cystic lesions in the liver. However, metastasis is a rare cause, and cystic metastasis from medullary thyroid carcinoma has not yet been reported. A 46-year-old Japanese man presented to our hospital with a mass in the left side of his neck. Neck and thyroid ultrasonography revealed a thyroid tumor with calcification and enlarged cervical lymph nodes. He had a family history of medullary thyroid cancer. Computed tomography revealed a tumor in the thyroid and multiple cysts in the liver. Total thyroidectomy with modified neck and upper mediastinum dissections were performed. After surgery, vandetanib treatment was initiated owing to tumor progression; following this, the liver cysts increased in size, abdominal distension appeared, and serum liver enzyme levels were found to be elevated. Percutaneous liver cyst puncture was performed to reduce abdominal distension; however, it was ineffective. The liver enzyme levels improved after replacing vandetanib with lenvatinib treatment. The liver cysts in this case were indicated to be associated with medullary thyroid carcinoma.

## 1. Introduction

Medullary thyroid carcinoma (MTC) accounts for 3%–5% of all thyroid malignancies [1, 2]. Approximately 75% of MTC cases are sporadic, while the remaining 25% are hereditary [2]. Metastases to the liver occur in 45% of patients with advanced MTC [1]; however, to the best of our knowledge, cystic metastasis from MTC has not been reported previously. Here, we report the case of MTC with liver cysts that increased in size during vandetanib treatment.

## 2. Case Presentation

A 46-year-old Japanese man presented to our hospital with a mass in his left neck. He had a palpable nodule in the thyroid and palpable left cervical lymph nodes. His anamnesis revealed irritable bowel syndrome. He had no previous history of thyroid disease or other endocrinopathy but had a family history of thyroid cancer; his mother and maternal uncle had thyroid cancer and pheochromocytoma, and a maternal cousin had MTC. Rearranged during Transfection (RET) mutation analysis was positive, and he was determined as having a RET mutation at codon 634 in exon 11.

**Figure 1:**
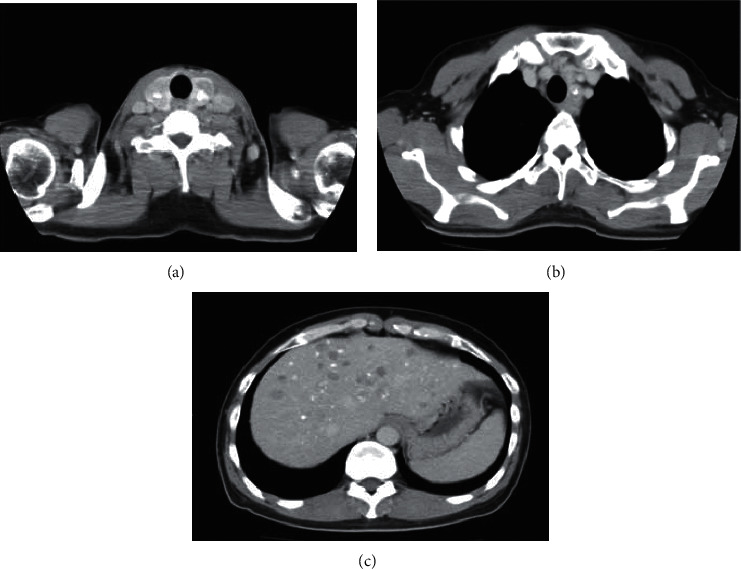
At the first visit to our hospital, computed tomography revealed a thyroid tumor (a), lymph node metastasis (b), and multiple cysts in the liver (c). The tumor was accompanied by calcification.

Ultrasonography of the neck and thyroid revealed a thyroid tumor with calcification and enlarged cervical lymph nodes. Serum carcinoembryonic antigen (CEA) and calcitonin levels were 2,540 ng/ml and 35,100 pg/ml, respectively. Computed tomography scans revealed a 2.5 cm × 3.5 cm thyroid tumor and multiple cysts in the liver. Calcifications were also observed in the thyroid, lymph node, and liver. No adrenal tumor was observed (Figure 1).

**Figure 2:**
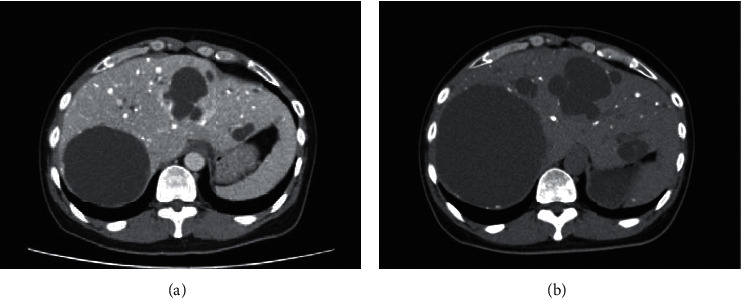
Computed tomography revealed that the liver cysts were increased in size after vandetanib treatment. (a) At 42 months after vandetanib treatment initiation. (b) At 48 months after vandetanib treatment initiation, abdominal distension developed.

**Figure 3:**
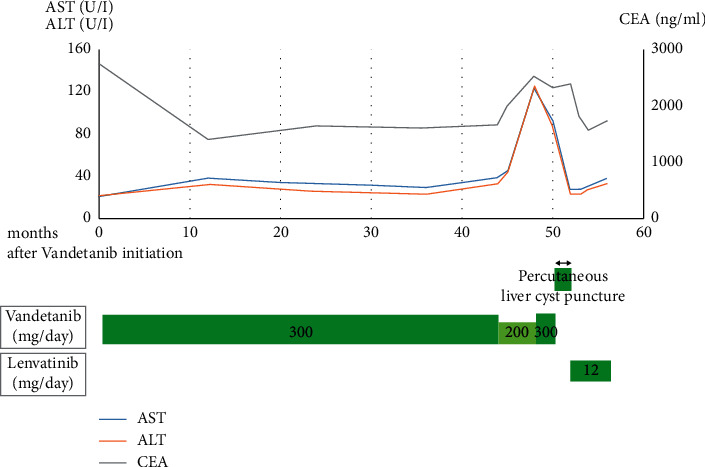
Clinical course and blood examination. AST: aspartate aminotransferase, ALT: alanine aminotransferase, and CEA: carcinoembryonic antigen.

Total thyroidectomy and modified neck and upper mediastinum dissections were performed. The pathologic diagnosis was medullary carcinoma (pT3N1bM1). Four months later, vandetanib treatment was initiated at a dose of 300 mg/day owing to progression of the liver metastasis. At 44 months after the initiation of vandetanib treatment, its dose was reduced to 200 mg/day owing to increased serum creatinine levels. After the dose reduction, the levels of CEA, calcitonin, and liver enzymes, such as aspartate aminotransferase (AST) and alanine aminotransferase (ALT), were found to be elevated. At 48 months after the initiation of vandetanib treatment, abdominal distension appeared, and the liver cysts increased to a maximum diameter of 14 cm (Figure 2). Thus, the dose of vandetanib was increased to 300 mg/day. Percutaneous liver cyst puncture was performed to reduce abdominal distension, and aspiration biopsy cytology of the liver cyst indicated atypia of undetermined significance. Although serum AST and ALT levels were transiently worse, the patient was discharged a month after the drainage. After discharge, he experienced gastrointestinal perforation associated with an ulcer, and emergency surgery was performed. The postoperative course was good, and he was discharged two weeks after the surgery. However, the liver cysts had increased in size and the serum CEA and calcitonin levels were elevated; we considered these as signs of disease progression. Thus, vandetanib treatment was discontinued, and lenvatinib treatment was initiated at a dose of 12 mg/day owing to increased serum AST and ALT levels. At 8 weeks after the initiation of lenvatinib treatment, the serum CEA and calcitonin levels were decreased to 1589.5 ng/ml and 83,800 pg/ml, respectively, while the serum liver enzyme levels were within the normal range (Figure 3).

## 3. Discussion

MTC is a type of neuroendocrine tumor that arises from neural crest-derived parafollicular C-cells [1], which secrete calcitonin and CEA [2]. Calcitonin is a very specific tumor marker, whereas CEA is a non-specific tumor marker used for MTC surveillance [2]. The serum concentrations of these markers are directly related to C-cell mass [1].

Tyrosine kinase inhibitors (TKIs) are used for the treatment of MTC. Two TKIs, vandetanib and cabozantinib, prolonged progression-free survival compared with placebo in phase III clinical trials [3, 4]. Based on these phase III clinical trials, the U.S. Food and Drug Administration and the European Medicines Agency approved vandetanib and cabozantinib for the treatment of patients with advanced progressive MTC [1]. In Japan, the corresponding agency approved vandetanib, sorafenib, and lenvatinib for the treatment of advanced progressive MTC [5].

In this case, vandetanib treatment was initiated because liver metastasis had progressed, following which the liver cysts considerably increased in size. At the beginning of the treatment, we assumed that this change was attributable to the effect of vandetanib treatment. However, the disease course indicated that liver dysfunction might have been caused by vandetanib. As the liver cysts had considerably increased in size and the serum CEA and calcitonin levels were elevated, we assumed that vandetanib was ineffective for this patient. Therefore, vandetanib was discontinued and replaced with lenvatinib.

After shifting from vandetanib treatment to lenvatinib treatment, the liver enzyme and tumor marker levels did not increase, which indicated that the liver dysfunction was caused by disease progression and that vandetanib was ineffective for this patient. According to a randomized, double-blind phase III trial, liver dysfunction is not a common adverse event of vandetanib treatment [3].

This patient had multiple liver cysts at initial presentation to our hospital. The incidence of cystic liver lesions is reported to be as high as 5% of the population [6]. The most common lesion type is benign simple cyst, and causes of cystic liver lesions include polycystic disease, parasite infection, abscess, or neoplasm. In cases of neoplasm, biliary cystadenoma and cystadenocarcinoma account for 5% of cystic lesions of the liver [7]. Cystic tumors, such as ovary and pancreatic cystadenocarcinomas, can metastasize to the liver. Although secondary neoplastic liver cysts are rare, cystic metastasis from gastrointestinal stromal tumors have been reported [8]. However, to the best of our knowledge, cystic metastasis from MTC has not been reported previously. In this case, despite initiating vandetanib treatment, the liver cysts increased in size and serum liver enzyme levels remained elevated. The cysts also caused abdominal distension; thus, we performed percutaneous liver cyst puncture. Aspiration biopsy cytology of liver cyst revealed atypia of undetermined significance as only few atypical cells were found. Moreover, we did not check the CEA and calcitonin level in the cystic fluid. Cyst puncture was ineffective, but after lenvatinib initiation, the liver cysts did not increase in size and serum liver enzyme levels improved owing to the antineoplastic effect of lenvatinib. Although the aspiration biopsy cytology of the liver cyst did not indicate metastasis, the cause of the liver cysts was believed to be the progression of the disease, as the cysts were found to be enlarged in the metastatic lesion and showed a tendency to improve with a change in the treatment.

This case indicated that cystic lesions of the liver may occur alongside MTC. It is also possible that increased liver cysts indicate disease progression, which may necessitate a change in treatment.

In conclusion, we reported the case of a patient with MTC and liver cysts that developed during vandetanib treatment. The liver cysts caused an increase in serum liver enzyme levels and abdominal distension. Percutaneous liver cyst puncture was ineffective, but treatment changes improved the serum liver enzyme, CEA, and calcitonin levels. This case indicated that MTC may occur along with liver cysts, and an increase in the size of the liver cysts can cause elevation in serum liver enzyme levels. When the liver cysts increase in size and liver enzyme levels are elevated, the treatment may need to be changed.

## Data Availability

The data used to support the findings of this work are included within the article.
